# Genome Sequencing of an Escherichia coli Sequence Type 617 Strain Isolated from Beach Ghost Shrimp (Callichirus major) from a Heavily Polluted Ecosystem Reveals a Wider Resistome against Heavy Metals and Antibiotics

**DOI:** 10.1128/MRA.01471-18

**Published:** 2019-01-17

**Authors:** Daniel F. Monte, Fábio P. Sellera, Miriam R. Fernandes, Quézia Moura, Mariza Landgraf, Nilton Lincopan

**Affiliations:** a Department of Food and Experimental Nutrition, Food Research Center, Faculty of Pharmaceutical Sciences, University of São Paulo, São Paulo, Brazil; b Department of Internal Medicine, School of Veterinary Medicine and Animal Science, University of São Paulo, São Paulo, Brazil; c Department of Clinical Analysis, Faculty of Pharmaceutical Sciences, University of São Paulo, São Paulo, Brazil; d Department of Microbiology, Institute of Biomedical Sciences, University of São Paulo, São Paulo, Brazil; University of Maryland School of Medicine

## Abstract

Here, we present the draft genome sequence of a multidrug-resistant (MDR) Escherichia coli strain belonging to sequence type 617 (ST617), isolated from beach ghost shrimp from polluted coastal waters in Brazil. These data provide valuable information for comparative genomic analysis, related to the dissemination of MDR E. coli in marine ecosystems.

## ANNOUNCEMENT

The growing occurrence of multidrug-resistant (MDR) Enterobacteriaceae in marine environments has been reported with particular concern about global public health (1). Recently, we reported the occurrence of Escherichia coli carrying clinically relevant resistance genes in recreational waters, seabirds, wild fishes, and bivalves in a heavily polluted Brazilian coastline (2–5), highlighting an urgent need to monitor marine environments. Here, we present the draft genome sequence of an MDR E. coli isolate (ECCO2) recovered from beach ghost shrimp (Callichirus major) in an impacted ecosystem.

Bacterial isolation and DNA extraction were performed as previously described (6, 7). Briefly, shrimp were captured using a specific slurp gun and then placed into sterile plastic bags (Whirl-Pak; Nasco, WI, USA). Samples (25 g) were dispensed in 225 ml of MacConkey broth and incubated at 37°C for 24 h. After incubation, an aliquot of 1 ml of MacConkey broth was serially diluted on buffered peptone water and inoculated onto MacConkey agar plates supplemented with ceftriaxone, meropenem, or colistin (2 µg/ml each; Sigma-Aldrich, St. Louis, MO) and incubated at 37°C for 24 h. A ceftriaxone-resistant E. coli isolate (ECCO2) was identified by matrix-assisted laser desorption ionization–time of flight mass spectrometry (MALDI-TOF). DNA extraction was performed using the PureLink quick gel extraction kit (Life Technologies, Carlsbad, CA). Next, DNA quality and quantity were checked with a NanoDrop spectrophotometer (Thermo Scientific) and Qubit 2.0 fluorometer (Life Technologies), respectively. Afterward, the genomic library was prepared using the Nextera XT DNA library preparation kit (Illumina, San Diego, CA) and subsequently sequenced using the Illumina NextSeq 550 platform, with 2 × 75-bp paired-end reads and a genome coverage of 290.0×.

Resulting FastQ data were imported to the CLC genomic workbench 10 (Qiagen). Raw reads (∼29 million) were inspected for quality evaluation, and a trimming/cleanup step was applied to avoid a contamination of reads with barcodes, adapter sequences, and a large presence of Ns before assembly. In this regard, the software has ensured that the read length is in the appropriate size of 300 bp and a G+C content of around 50% to ensure accurate assembly. Finally, reads were *de novo* assembled using default settings in CLC workbench (such as automatic word [20] and bubble size [50]), a minimum contig length of 200 nucleotides, auto-detect paired distances, and mapping reads back to contigs.

The generated assembly showed a total of 5,133 genes with 5,060 protein-coding sequences. A total of 197 contigs were obtained, with an *N*_50_ value of 86,325 bp, as well as a G+C content of 50.6%, which was annotated by the NCBI Prokaryotic Genome Annotation Pipeline (PGAP) (https://www.ncbi.nlm.nih.gov/genome/annotation_prok/). The genome of ECCO2 was 4.8 Mb in size, containing 56 tRNAs, 2 rRNAs, 9 noncoding RNAs (ncRNAs), 284 pseudogenes, and 2 CRISPR arrays.

The resistome, plasmid replicons, multilocus sequence type (MLST), serotype, and virulome were identified using ResFinder 3.1, PlasmidFinder 2.0, MLST 2.0, SerotypeFinder 2.0, and VirulenceFinder 2.0 databases (95% of identity and 60% of minimum length), respectively (http://genomicepidemiology.org/). In this regard, the resistome revealed the presence of genes conferring resistance to β-lactams (*bla*_CMY-2_ and *bla*_TEM-1B_), quinolones (*qnrS1*), aminoglycosides [*aph*(*6*)*-Id* and *aph*(*3″*)*-Ib*], tetracycline (*tetB* and *tetD*), sulfonamide (*sul2*), and trimethoprim (*dfrA8*). In fact, ECCO2 displayed an MDR profile to cefoxitin (MIC, >32 µg/ml), enrofloxacin (MIC, 16 µg/ml), levofloxacin (MIC, 16 µg/ml), nalidixic acid (MIC, 32 µg/ml), ciprofloxacin (MIC, 4 µg/ml), amikacin (MIC, 32 µg/ml), gentamicin (MIC, 16 µg/ml), tetracycline (MIC, 32 µg/ml), and trimethoprim-sulfamethoxazole (MIC, 4 µg/ml), determined by disc diffusion and Etest methods (8). Additionally, chromosomal point mutations were detected in *gyrA* (S83L), *gyrB* (D87N), and *parC* (S80I) genes, which confer resistance to fluoroquinolones (9). Furthermore, genes conferring resistance to quaternary ammonium compounds (*sugE*) and heavy metals (silver, *silR7*) were also identified. The ECCO2 isolate was assigned to the serotype O89:H9, and virulome analysis detected *gad* (glutamate decarboxylase) and *iss* (increased serum survival) genes.

IncFIB, IncFIC, IncFII, and IncX3 incompatibility group plasmids were identified. Genomic analysis confirmed the presence of the *bla*_CMY-2_ gene on the IncFII plasmid. MLST analysis assigned the ECCO2 strain to sequence type 617 (ST617). ST617 belongs to clonal complex 10 (CC10), which is widespread internationally and related to clinical strains found in environmental, human, and food samples, and mostly in association with a broad-spectrum cephalosporin-resistant phenotype promoted by the acquisition of plasmid-mediated *bla*_CMY-2_ and *bla*_CTX-M_-type genes (1, 2, 6, 10–12). In this regard, extended-spectrum β-lactamase (CTX-M)-producing E. coli ST617 has been reported in urban lakes in Brazil (11) and in Franklin’s gulls (Leucophaeus pipixcan) in Chile (12). Therefore, polluted environments could favor bacterial transmissions to wildlife (1, 2, 12).

In summary, we present the first draft genome sequence of an MDR E. coli strain displaying an extended resistome and recovered from beach shrimp. Our findings suggest that benthic animals living on polluted sand bottoms could become new hosts and vehicles for transmissions of MDR bacteria to marine predators, adding valuable information in the routes of transmission of MDR E. coli in the marine environment. Therefore, genomic surveillance studies in coastal habitats are urgently required.

### Data availability.

This whole-genome shotgun project has been deposited at DDBJ/ENA/GenBank under the accession number PIZJ00000000. The described version used in this paper is PIZJ01000000, and the SRA run number is SRR8186051.
